# Machine Learning-Based Framework for Differential Diagnosis Between Vascular Dementia and Alzheimer's Disease Using Structural MRI Features

**DOI:** 10.3389/fneur.2019.01097

**Published:** 2019-10-25

**Authors:** Yineng Zheng, Haoming Guo, Lijuan Zhang, Jiahui Wu, Qi Li, Fajin Lv

**Affiliations:** ^1^Department of Radiology, The First Affiliated Hospital of Chongqing Medical University, Chongqing, China; ^2^Department of Neurology, The First Affiliated Hospital of Chongqing Medical University, Chongqing, China

**Keywords:** structural MRI, VaD and AD, SVM, machine learning, computer-aided diagnosis

## Abstract

**Background and Objective:** Vascular dementia (VaD) and Alzheimer's disease (AD) could be characterized by the same syndrome of dementia. This study aims to assess whether multi-parameter features derived from structural MRI can serve as the informative biomarker for differential diagnosis between VaD and AD using machine learning.

**Methods:** A total of 93 patients imaged with brain MRI including 58 AD and 35 VaD confirmed by two chief physicians were recruited in this study from June 2013 to July 2019. Automated brain tissue segmentation was performed by the AccuBrain tool to extract multi-parameter volumetric measurements from different brain regions. Firstly, a total of 62 structural MRI biomarkers were addressed to select significantly different features between VaD and AD for dimensionality reduction. Then, the least absolute shrinkage and selection operator (LASSO) was further used to construct a feature set that is fed into a support vector machine (SVM) classifier. To ensure the unbiased evaluation of model performance, a comparative study of classification models was implemented by using different machine learning algorithms in order to determine which performs best in the application of differential diagnosis between VaD and AD. The diagnostic performance of the classification models was evaluated by the quantitative metrics derived from the receiver operating characteristic curve (ROC).

**Results:** The experimental results demonstrate that the SVM with RBF achieved an encouraging performance with sensitivity (SEN), specificity (SPE), and accuracy (ACC) values of 82.65%, 87.17%, and 84.35%, respectively (AUC = 0.861, 95% CI = 0.820–0.902), for the differential diagnosis between VaD and AD.

**Conclusions:** The proposed computer-aided diagnosis method highlights the potential of combining structural MRI and machine learning to support clinical decision making in distinction of VaD vs. AD.

## Introduction

Dementia is a typical clinical syndrome of cognitive decline that interferes with the ability to perform daily activities (1) and occurs due to physical changes of brain structure and function. It is a progressive disease, indicating that it gets worse over time in terms of memory loss, cognitive dysfunction, and behavior. It is reported that the number of patients affected by dementia is believed to be close to 60 million people in 2018, and this number will almost reach 75 million in 2030 and triple with 130 million in 2050. Alzheimer's disease (AD) and vascular dementia (VaD) are the first and second most common forms of dementia, respectively (2). They have several symptoms, pathophysiology, and comorbid clinical manifestation that overlap that make them difficult to distinguish.

At present, the differential diagnosis between AD and VaD is still largely based on clinical guidelines with the exception of the exclusion of other diseases that are able to result in dementia. VaD is usually diagnosed through the combination of neurological examination, cognitive functioning tests, and brain scanning techniques (3). AD is often diagnosed by excluding other causes rather than being able to pinpoint the diagnosis through imageological or biochemical examination (4, 5). Conventional magnetic resonance imaging (MRI) could only discover certain clinical entities such as vascular changes and stroke or ischemic attack occurring in a specific area of brain (6), suggesting their association with VaD. Molecular neuroimaging technique plays an important role in the diagnosis of dementia (7), but it is difficult to determine the types of dementia. However, as is the case with AD, a definite diagnosis of VaD can only be made by brain autopsy. Although some similar cognitive examinations are employed to evaluate brain function (8), there is no test to diagnose AD at this time, so neurological physicians generally rule out other reversible causes of confusion such as normal pressure hydrocephalus, as well as other types of dementia or delirium.

Under attack by AD, the structure changes of temporal lobe, hippocampus, and entorhinal cortex may be changed firstly (4, 9). Hill et al. (10) suggested that structural MRI biomarkers could facilitate the clinical trials of AD. Moreover, several machine learning-based studies have successfully classified and predicted AD and mild cognitive impairment (MCI) using structural MRI features (11–13). It indicates that the changes of structural MRI are sensitive indicators for dementia, but there were no previous studies referring to the distinction between VaD and AD.

**Figure 1:**
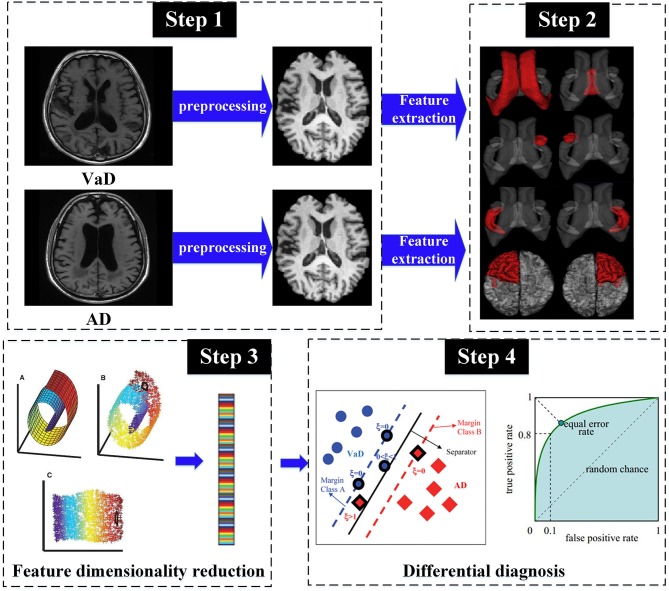
The pipeline of proposed framework for the distinction of VaD vs. AD.

A series of studies mentioned above suggest that volumetric measurements of brain MR images are generally a significative type of biomarker. However, to our knowledge, whether multi-parameter structural MRI features can serve as the informative biomarker to detect the differences between VaD and AD is unknown, and little work has been done on machine learning to distinguish VaD from AD. Therefore, despite recent developments in the detection of AD, differential diagnosis between AD and VaD is still challenging and requires further investigation. It is critically important to find a way to be potentially capable of differentiating VaD from AD. In this study, we present a support vector machine (SVM)-based machine learning framework in combination with a range of volumetric measurements of different brain tissues to provide clinical information for differential diagnosis between VaD and AD (Figure 1).

## Materials and Methods

### Subjects and Inclusion Criteria

This retrospective study was approved by the Ethics Committee of the First Affiliated Hospital of Chongqing Medical University. The written full informed consents were obtained from all subjects. From June 2013 to July 2019, 122 patients imaged with brain MRI who were hospitalized in the First Affiliated Hospital of Chongqing Medical University were recruited for this study. The diagnosis was confirmed by two chief physicians. VaD patients fulfilled the criteria of NINDS-AIREN (3) and AD patients fulfilled the criteria of NINCDS-ADRDA (14). Exclusion criteria are as follows: (1) patients have both AD and VaD (*n* = 8); (2) patients with suboptimal image quality because of head motion or susceptibility artifacts (*n* = 14); and (3) the areas of hyperintensities are so large that they have little influence on the segmentation accuracy of AD and VaD (*n* = 7). Finally, 58 AD patients and 35 VaD patients were enrolled and the demographic data are summarized in Table 1. Sex ratio and age distribution did not differ significantly between both groups (χ^2^ test, *P* = 0.73 and Wilcoxon rank-sum test, *P* = 0.24).

**Table 1:** Demographic information.

	**Patients with VaD**	**Patients with AD**	***P*-value**
Number	35	58	
Female/male	16/19	30/28	0.73
Age	72.74 ± 10.19	70.33 ± 9.18	0.24
MMSE	21.79 ± 5.31	22.61 ± 4.87	0.29

### MRI Acquisition

All subjects underwent multisequence imaging protocol on a 1.5-T MRI scanner (MAGNETOM ESSENZA, Siemens Healthineers, Germany and Signa HDxt, GE Healthcare, USA). For each patient, two sequences were collected in our study: (1) T1-weighted spin-echo (T1W) image: repetition time/echo time (TR/TE) = 1800/22 ms; matrix size = 512 × 512; field of view (FOV) = 240 × 240 mm^2^; slice thickness = 3 mm; gap = 1.5 mm; (2) T2 fluid attenuated inversion recovery (T2 FLAIR) image: TR/TE = 8000/120 ms; matrix size = 512 × 512; field of view (FOV) = 240 × 240 mm^2^; slice thickness = 3 mm; gap = 1.5 mm.

### Image Pre-processing

Firstly, T1W and T2 FLAIR images are skull-stripped, performed using the FMRIB software library (http://fsl.fmrib.ox.ac.uk/fsl/fslwiki/FSL). Then, the skull-stripped T2 FLAIR images were aligned and registered to T1WI images using SPM12 based on rigid transformation and normalized mutual information (15). After the above operations, N4 bias correction was performed on T1W and T2 FLAIR images to remove low-frequency intensity non-uniformity (http://stnava.github.io/ANTs/).

### MRI Structure-Based Feature Extraction and Selection

In our study, multi-parameter structural MRI indexes were used as the feature set to train and test machine learning model. A reliable and robust automated software AccuBrain (BrainNow Medical Technology Limited, Hong Kong, China) performs brain structure and tissue segmentation to obtain multiple volumetric measurements of different brain substructures and subcortical tissues (16). It could provide the quantitative volumetry of memory-related cerebral areas in a fully automatic mode. After feature extraction, the next step is construction of the optimal feature subset. As feature selection is an important problem for pattern classification that has become an apparent need in machine learning, the effectiveness of features is directly associated with the performance of classifier. Firstly, normality and homogeneity of variance have been examined by Kolmogorov–Smirnov test and Levene test, respectively, and features with skewed distribution or normal distribution have been compared using the Mann–Whitney *U* test or independent Student *t* test to select certain volumetric indexes with significant difference (*P* < 0.05) as the representative features (17). Then, the least absolute shrinkage and selection operator (LASSO) method was used for the selected features to form the fusion feature signature (feature subset). The feature selection methods were performed with the R software (version 3.5.1; http://www.R-project.org).

### Machine Learning Modeling and Performance Evaluation

The typical kernel algorithms in machine learning such as SVM were employed to identify VaD from AD. Based on structural risk minimization, SVM classifier finds an optimal separating hyperplane with maximum margin to distinguish VaD from AD in the corresponding high-dimension feature space mapped by the input feature subset (18). In this study, we used the LibSVM toolbox (version 3.22) for the implementation of SVM classifier with linear and radial basis kernel functions (19). In addition, genetic algorithm (GA) was conducted to select the optimal parameters of the LibSVM classifier. For performance comparison of classification, we have adopted different machine learning algorithms such as K-nearest neighbor (KNN), logistic regression (LR), and random forest (RF) to test which model performs best in differentiating between VaD and AD, compared with SVM. A brief overview about the corresponding parameters of the classifiers is given in Table 2.

**Table 2:** The implementation details of the different classifiers.

**Classifiers**	**Parameter setting**
KNN	20 different values of number of neighbors range from 2 to 21
LR	Penalty: L1, Tol = 0.0001, C = 1.0, Max_ iteration = 500
RF	Ntree = {100, 200, 300, 400, 500}, Mtr = [2:2:50]
SVM	Population_size = 50, Iteration = 1,000, Pc = [0.4, 0.99], Pm = [0.0001, 0.1], Kernel parameter = {10^−2^, 10^−1^, 10, 10^2^}

*KNN, K-nearest neighbor; LR, logistic regression; RF, random forest; SVM, support vector machine*.

**Figure 2:**
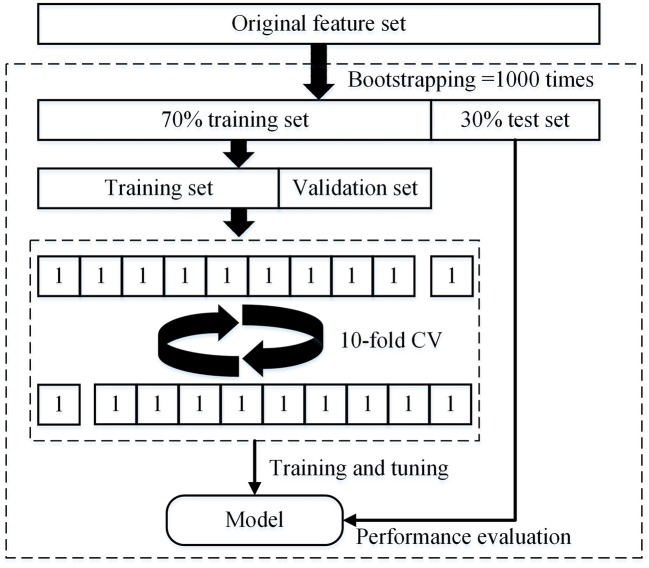
Structure of the nested 10-fold cross-validation for evaluating the performance of machine learning models.

In this study, the dataset was divided into two portions called training set and testing set, 70% of which were used as training set, and the remaining 30% were used as test set. In the training set, we used the 10-fold cross-validation (CV) to train and tune the model. The training set was divided into 10 subsets, each as a verification set for monitoring and tuning the parameters of training process, and the other 9 subsets was used for training the model. The test set was used only to assess the performance of the model. In addition, a bootstrap resample method (1,000 times) was used to decrease the bias of overfitting and evaluate the robustness of each diagnostic model. Hence, the accuracy (ACC), sensitivity (SEN), specificity (SPE), and area under the curve (AUC) of model are calculated by taking the average of the results of 1,000 times tests. The detailed procedure of parameter tuning and performance testing is shown in Figure 2.

## Results

This section presents the experimental results obtained through the quantitative volumetry of structural MRI using AccuBrain software on T1W and T2 FLAIR imaging, as efficient biomarkers disclosing the significant change of volumes in the memory-related cerebral areas between VaD and AD. On the other hand, the classification performance obtained with or without the feature selection method was compared and analyzed.

### Differences in the Volumetric Features of Different Brain Tissues Between VaD and AD

The result presentation of quantitative volumetry in structural MRI was reported for the following cases: (1) volume differences of memory-related cerebral areas between patients who suffer from VaD and AD, (2) symmetry of brain substructures between the patients with VaD and AD, and (3) volume atrophy differences between VaD and AD (Table 3). The structural changes of memory-related cerebral areas were obvious and significant between the patients with VaD and AD, while volume differences could have potential to differentiate VaD from AD. When compared to the AD patients, the VaD patients show significantly higher volume values (*P* < 0.05) in brain parenchyma, hippocampus, amygdala, and accumbens nucleus, and significantly lower volume values (*P* < 0.05) in pallidum. The significant difference for the symmetry of certain brain substructures has also been observed between VaD and AD such as hippocampus, amygdala, caudate, pallidum, and accumbens nucleus (*P* < 0.05). The group comparison reveals a significant decline in the volume values of bilateral frontal lobe, occipital lobe, temporal lobe, and parietal lobe as well as significant volume atrophy differences in occipital lobe and parietal lobe in the patients with AD (*P* < 0.05).

**Table 3:** The structural MRI features with statistical differences between patients with VaD and AD.

	**Brain substructures/regions**	**Volume (ml)**		
		**VaD**	**AD**	***P*-value**
Brain substructures	Hippocampus	5.62 ± 0.79	5.13 ± 0.74	**0.001**
	Amygdala	3.37 ± 0.49	2.63 ± 0.27	**0.001**
	Pallidum	2.23 ± 0.31	2.49 ± 0.51	**0.031**
	Accumbens nucleus	0.77 ± 0.14	0.68 ± 0.11	**0.003**
Symmetry of brain substructures	Hippocampus (L)	2.90 ± 0.53	2.47 ± 0.42	**0.004**
	Hippocampus (R)	2.87 ± 0.42	2.66 ± 0.31	**0.001**
	Amygdala (L)	1.46 ± 0.27	1.19 ± 0.21	**0.001**
	Amygdala (R)	1.69 ± 0.34	1.39 ± 0.25	**0.001**
	Caudate (L)	3.07 ± 0.51	2.88 ± 0.49	**0.028**
	Pallidum (L)	1.21 ± 0.58	1.29 ± 0.31	**0.031**
	Accumbens nucleus (L)	0.36 ± 0.06	0.32 ± 0.06	**0.012**
	Accumbens nucleus (R)	0.39 ± 0.06	0.35 ± 0.06	**0.013**
Symmetry of brain regions	Frontal lobe (R)	65.14 ± 9.94	62.17 ± 7.86	**0.011**
	Occipital lobe (L)	34.51 ± 6.46	31.28 ± 6.39	**0.001**
	Occipital lobe (R)	31.08 ± 4.97	28.86 ± 5.42	**0.003**
	Temporal lobe (L)	45.46 ± 5.34	43.29 ± 6.47	**0.001**
	Temporal lobe (R)	46.26 ± 6.51	42.93 ± 5.86	**0.002**
	Parietal lobe (L)	36.88 ± 5.75	30.73 ± 4.76	**0.001**
	Parietal lobe (R)	38.12 ± 6.96	31.45 ± 6.85	**0.002**
	Insular (R)	5.67 ± 1.13	5.06 ± 1.07	**0.045**

*Bold values indicate the statistically different*.

### Performance Comparison of Machine Learning Models for Differential Diagnosis

**Figure 3:**
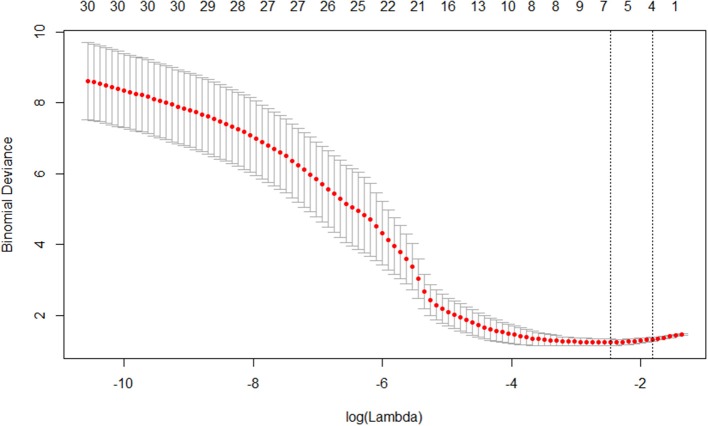
Plot of coefficients-lambda obtained by LASSO.

**Figure 4:**
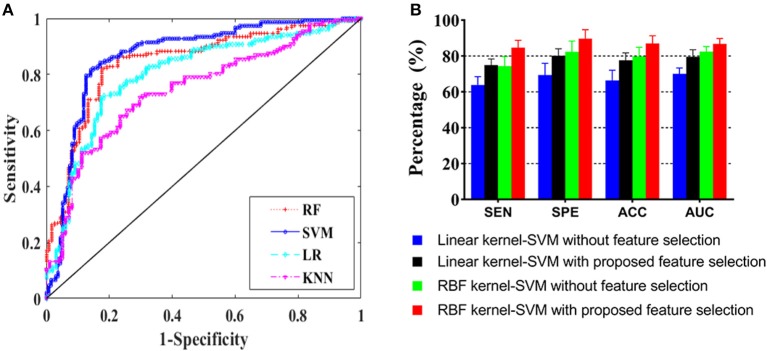
Model performance presentation. **(A)** ROC curves for different machine learning models and **(B)** performance comparison for SVM with linear and radial base function (RBF) kernels on the raw and optimized datasets.

This section presents the results of comparing differential diagnosis between VaD and AD obtained by the different machine learning models. The 20 significantly different features (Table 3) selected from 62 quantified structural MRI measures obtained by AccuBrain software were ranked by LASSO feature selection. Then, five top-ranked features were selected as the input of machine learning model (Figure 3). GA was employed to find the global optimum solution of SVM, the parameters of which were set as follows: population size = 50, iteration times = 1,000, probability of crossover = 0.6 and probability of mutation = 0.1. Table 4 presents the accuracy rates and the corresponding AUC values of different machine learning models in training, verification, and test set. The overall result of performance comparison is shown in Figure 4A. It demonstrated that SVM could achieve more encouraging performance than other frequently used classifiers that are suitable for small datasets in the application of differentiating VaD from AD. The result obtained by SVM classifiers with different kernel functions (linear and RBF kernels) on the raw feature set and the optimal feature subset addressed through the feature selection method is shown in Figure 4B. This indicates that the SVM with RBF kernel generally yields higher performance metrics and is more flexible than that with linear kernel, and the combining of machine learning and feature selection can increase the classification performance of the model. The confusion matrix of the proposed SVM model in a single experiment is shown in Table 5. When compared to classification using raw features and the other machine learning models, classification by the selective features and the proposed SVM model improves the accuracy rate to a significant level, which indicates a powerful performance in differential diagnosis between VaD and AD.

**Table 4:** The result of different machine learning models in training, verification, and test set.

	**Training set**		**Verification set**		**Test set**	
	**Accuracy (%)**	**AUC**	**Accuracy (%)**	**AUC**	**Accuracy (%)**	**AUC**
KNN	72.63	0.737	70.59	0.722	68.14	0.691
LR	77.14	0.789	74.96	0.754	73.62	0.747
RF	82.89	0.833	83.65	0.845	81.17	0.829
SVM	86.47	0.887	84.71	0.868	84.35	0.861

**Table 5:** The confusion matrix of SVM model in a single experiment.

		**Truth**		**Recall**
		**AD**	**VaD**	
Prediction	AD	48	4	0.828
	VaD	10	31	0.886
	Precision	0.923	0.756	0.849

## Discussion

Many recent neuroimaging studies have focused on the use of advanced machine learning algorithms to solve problems in differential diagnosis, especially cases in the combination of medical science and engineering, such as the identification of the early stage of AD (20) and distinguishing between MCI and AD (21).

VaD characterized by the syndrome of intellectual disability such as hypomnesia and impairment of daily activities or living is very similar to AD. Therefore, accurate differential diagnosis is essential to receive timely clinical treatment that delays the progression of disease (22), which is helpful and required. Research on the biomarkers for differential diagnosis between VaD and AD mainly includes biochemical, genomics, proteomics, and neurophysiology markers such as neurofilament light unit and neurofilament protein levels (23), the ratio of plasma Aβ-38/-40 peptides (24), and phosphorylated tau proteins (25). Moreover, another critical requirement for clinical application is in the pursuit of neuroimaging biomarkers. In our study, a machine learning model derived from an RBF SVM classifier combined with LASSO and GA has been proposed for the differential diagnosis between VaD and AD. To our knowledge, this study is a unique combination of structural MRI biomarkers and machine learning for the differentiation of VaD vs. AD not used before.

Abrigo et al. (16) have affirmed that AccuBrain software could accurately and reliably segment hippocampus in accordance with the EADC-ADNI protocol so as to ensure the robustness, reproducibility, and reliability of structural MRI measurements such as volume, structural symmetry, and atrophy. The present result indicates that the symmetry differences and volume differences of hippocampus, amygdala, accumbens nucleus, and pallidum could have potential to differentiate VaD from AD (10, 26). We found that the involvement of brain regions is a relevant association between the volumetric change or atrophy identified in AD vs. VaD and that identified in AD vs. MCI (27). Because VaD is one of the main categories of senile dementia, the brain structure changes in the patients with VaD are similar to those with AD, which is in accordance with our current findings. Cuingnet et al. (28) found that the main brain area for the differentiation between AD and healthy individuals was the medial temporal lobe, similar to our study.

Our study indicates that the structural MRI measurements could be considered as core biomarkers for the differentiation of not only MCI vs. AD but also VaD vs. AD, combined with other researches (29, 30). It is manifested that the volumetric MRI studies of cerebral areas related with learning and memory such as hippocampus, amygdala, and accumbens nucleus are associated with neurodegeneration and shown to be sensitive to dementia severity (31). The hippocampus is the top-ranked effective indicator for differential diagnosis, which is in agreement with a previous study on the correlation between hippocampal volume and severity degree of dementia (32). Tondelli et al. (33) have suggested that the structural MRI changes occur before cognitive decline in the patients with AD and could potentially detect the regions affected by AD neuropathology. However, it seems possible to detect a similar distribution of brain atrophy accompanied by cognitive disorder caused by alternative VaD or AD. This could potentially explain why many VaD patients were misdiagnosed as AD subjectively by young physicians in the clinic. VaD could be easily confused with AD, especially in the early stages (34, 35). It is believed that the combination of such diverse structural MRI biomarkers containing more of the information in MR scanning contributes to an accurate differentiation between VaD and AD, in comparison with a single biomarker. Machine learning algorithms have been validated to overcome this obstacle. SVM outperforms all other classification methods such as KNN, LR, decision-making trees, and RF in small-sample research. Our results point in the same direction, since the input measures used are very small datasets.

In our experiment, the diagnostic performance of SVM classifier is superior to that of the other machine learning models. This is probably because SVM is trained based on a convex optimization problem so as to obtain a global optimum solution (18). Compared with RF, it is more likely to model more functions with the kernel-based method and reach an optimal separating structure in a small sample training set as well as avoiding suffering from local minimum mistakes and overfitting. In addition, KNN tends to perform very well with a lot of data points and RF is inherently suited for multiclass problems, while SVM is intrinsically constructed for binary classification, and the latter is more suitable to the task of this study. Compared with LR, SVM minimizes hinge loss while LR minimizes logistic loss. This makes LR more sensitive to outliers because logistic loss diverges quicker than hinge loss. Besides, even though the data are distinguished sufficiently confidently, logistic loss does not reach zero. This might give rise to minor degradation in accuracy. The factors mentioned above support the fact that SVM outperforms the other classifiers in our study.

Some limitations in this study should be considered. First, our study only involved single-center data, and the small sample set was used, especially for VaD data. Transfer learning is an alternative method for the problem of lack of data (36). It is regarded as the use of a pre-trained model as a feature extractor, and then training and testing the classifier using the features that can be derived from fine-tuning the pre-trained model using source data. This will take the place of AccuBrain software as feature extractor. However, one of the purposes of the present study is to confirm that the AccuBrain software could effectively and reliably provide volumetric measurements of structural MRI as independent indicators, which helps to quantify the architectural differences and facilitates computer-aided diagnosis between AD and VaD. So, for this purpose, we did not consider using transfer learning in this study. Nevertheless, further expanding samples and launching multi-center studies in future work, transfer learning could be the best choice. Second, only the indicators from structural MRI are used. Next, a larger number of datasets including multi-center data could be applied to extend our study by combining other MRI-based biomarkers, such as functional parameters (DTI metrics), radiomics features, and brain connectome, which target subtler information. We have reasons to believe that the combination of structural MRI-based volumetric measurements and other markers would improve the degree to which structural features are sensitive to differentiation between VaD and AD.

## Data Availability Statement

All datasets generated for this study are included in the manuscript.

## Ethics Statement

This retrospective study was approved by the Ethics Committee of our hospital. The written full informed consents were obtained from all subjects.

## Author Contributions

YZ, LZ, QL, and FL designed the study. HG, JW, and QL collected patient data and provided clinical expertise. YZ processed and analyzed the MRI data, and drafted the manuscript. FL and QL interpreted the data for the work. All the authors approved the final version of the manuscript.

### Conflict of Interest

The authors declare that the research was conducted in the absence of any commercial or financial relationships that could be construed as a potential conflict of interest.
